# Field Margins, Foraging Distances and Their Impacts on Nesting Pollinator Success

**DOI:** 10.1371/journal.pone.0025971

**Published:** 2011-10-03

**Authors:** Sean A. Rands, Heather M. Whitney

**Affiliations:** 1 Centre for Behavioural Biology, School of Veterinary Science, University of Bristol, Bristol, United Kingdom; 2 School of Biological Sciences, University of Bristol, Bristol, United Kingdom; University of Arizona, United States of America

## Abstract

The areas of wild land around the edges of agricultural fields are a vital resource for many species. These include insect pollinators, to whom field margins provide both nest sites and important resources (especially when adjacent crops are not in flower). Nesting pollinators travel relatively short distances from the nest to forage: most species of bee are known to travel less than two kilometres away. In order to ensure that these pollinators have sufficient areas of wild land within reach of their nests, agricultural landscapes need to be designed to accommodate the limited travelling distances of nesting pollinators. We used a spatially-explicit modelling approach to consider whether increasing the width of wild strips of land within the agricultural landscape will enhance the amount of wild resources available to a nesting pollinator, and if it would impact differently on pollinators with differing foraging strategies. This was done both by creating field structures with a randomised geography, and by using landscape data based upon the British agricultural landscape. These models demonstrate that enhancing field margins should lead to an increase in the availability of forage to pollinators that nest within the landscape. With the exception of species that only forage within a very short range of their nest (less than 125 m), a given amount of field margin manipulation should enhance the proportion of land available to a pollinator for foraging regardless of the distance over which it normally travels to find food. A fixed amount of field edge manipulation should therefore be equally beneficial for both longer-distance nesting foragers such as honeybees, and short-distance foragers such as solitary bees.

## Introduction

Globally, evidence is accumulating that populations of both wild and managed pollinators are in decline [1]–[5]. Pollinator loss, particularly of bees, has drastic economic effects [6]–[8], and much effort has been put into both identifying the factors causing these losses, and attempting to reverse the declines [5], [9]–[11]. With increasingly large areas of land being used to grow single crops, the resulting loss of structural diversity within the landscape has been suggested to be a key contributory factor to pollinator loss [12], [13]. One strategy for reversing the decline land-use changes are causing is to add heterogeneous ‘refuge’ areas within the landscape. National and international agri-environment schemes offer subsidies to farmers for adding different forms of wildlife refuges within the agricultural landscape [9], [14], which include both leaving fields fallow, and adding set-aside ‘wild’ regions at the edges of fields, which aim to enhance the connectivity between natural areas and the land used for agriculture.

Field edges consist of a wide diversity of different sub-habitats, and include landscape features such as hedgerows, ditches, wooded areas, and stream edges [15], [16]. All of these have positive effects in enhancing the amount of local biodiversity [17]–[20]. Both floral [21], [22] and invertebrate biodiversity [23]–[29] are increased by field margins, including species that are natural enemies of crop pests [30]. Most importantly for pollination, field edges are attractive to foraging native bees [20], [31], [32], and increasing their width or the bank of floral resources within them has positive effects upon pollinator presence [27], [33], [34]. Therefore, as a remediation strategy, field edges could be manipulated in many different ways, such as enhancing their wild flower seedbank, or altering their spatial scale within and between fields. Since a given area of land cannot be used both for crops and as a wild refuge, farmers choosing to add wild land face a trade-off between the services provided by wild land and the costs of not using the land for direct production. Therefore, exploring how wild land such as field edges can be used to the best advantage is crucial for successfully implementing them within intensive agricultural systems.

Identifying how best to allocate wild land within an agricultural environment involves identifying how the organisms using the land will respond to environmental manipulations. If we assume that bees nest within minimally disturbed wild patches, it follows that their interaction with the local environment will be limited by the distance that they will typically forage around their nest. Many studies have attempted to quantify the maximum range over which the many species of bee (sampled from across the Apidae) will forage away from their nest *(e.g.* [35]–[46]). With the exception of the honeybees *Apis* spp., most of the Apidae appear to forage a maximum of 2km from their nests, and a majority of these forage under 1km [47], although they may not forage in the immediate range around their nest [48]. Therefore, if we are interested in addressing how the supplementation of field edges affects bee foraging success, we need to consider what type of land a manipulation makes available within this local area. If a particular species has a short foraging range, adding a given proportion of field edges into the local environment may have a different effect than it would to a longer-range forager, as such short foraging distance could be heavily affected by local landscape geometry.

Rands & Whitney [49] described a simple simulation model that explored the effects of field edge structure upon foraging success in bees. The quality of habitat available to a forager with a fixed foraging radius was considered, where the environment had a simplified grid-like structure. This model asked whether landscape structure could have an effect upon the availability of wild or crop forage to bees, dependent upon the bees' degree of constancy to the most abundant resource available (‘neophobia’, also discussed in [50], [51]). If agricultural practices mean that bees that are foraging in a landscape filled with large fields are over-attracted to single dominant crop type, the over-representation of a single source of resource in the bees' diet could have detrimental effects upon development of the colony [52], [53], as the lack of dietary diversity may lead to a lack of micro-nutrients essential to larval development. Rands & Whitney demonstrated that both the density of wild flowers within the field edge and the width of field edges relative to agricultural fields were important for enhancing resources available to bees. This became increasingly important if the bees showed some degree of preference for the most common resource available within the environment.

Although possibly representative of large-scale farms where the landscape structure is primarily homogeneous crop interspersed with very rare straight field edges, the grid-like structure of the fields considered by Rands & Whitney were arguably too artificial for most landscapes in Western Europe, where hedgerows and field margins are an integral part of the landscape. Here, we consider the effects of field edge manipulation upon the availability of wild area to bee-like foragers who are constrained by a need to return to a nest located within the field edges (which would make this comparable to economic models of central place foraging, *e.g.* [54]–[56]). Rands & Whitney [49] only considered changes in individual preference for monocultures in response to landscape manipulation. Here, we instead explore the overall availability of resources with respect to the flight distance of a nesting foraging and the degree to which the environment is manipulated. We consider both a landscape composed of randomly structured fields, and one composed of field forms that are extracted from British mapping, which we propose as a model system for exploring how the techniques could be used to consider any agricultural environment where landscape structure is known. As well as addressing whether the addition of space within field edges has an effect upon what is available to foragers, we also consider whether these effects vary for foragers who travel differing distances from their nest.

## Methods

### Models using a Voronoi-like randomised landscape

**Figure 1:**
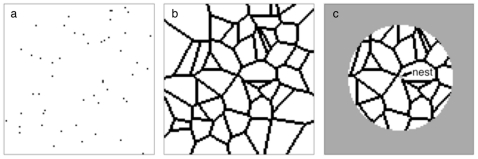
Simplified illustration of the Voronoi-like field generation process. Here, a 101×101 unit arena is populated with 50 field seeds that are randomly placed on unit cells (*a*). The nearest seed is calculated for all the cells within the arena (with random allocation if several seeds are equally closest). If a cell possesses at least one neighbour that does not share its nearest seed, it is designated an ‘edge’ cell, whereas cells where all four neighbours share the same nearest seed are designated ‘field’ cells: (*b*) shows black edge and white field cells for the field seeds given in (*a*). To calculate wild edge cell availability nest sites are randomly placed on edge cells, and the numbers of edge and field cells are tallied within a given foraging radius of the nest (the area within the grey circle in *c*).

All simulations took place within a 500×500 grid of unit squares (figure 1 gives a simplified illustrative version of the process on a reduced grid). Fields were created by selecting a predefined number of seeds at randomly selected points within the grid: all the points were randomly selected non-integer coordinates, and no more than one point occurred within any unit cell within the grid. Each of these seeds was given a unique identifier, and then each cell within the grid was then labelled with the identifier of the seed closest to its centre (or, in the unlikely event of several seeds being equally closest, one of these seeds was randomly selected as being the closest). Field edges were then defined as the cells whose four touching neighbours included at least one cell that did not share the same label as the target cell. This gave a structure based on a Voronoi tessellation, implemented within a discretised grid.

For simulations where field edge width was set as 1, only the cells determined above were considered to be edges. If edge width was 2, all the cells immediately connected to previously-defined field edge cells were considered to be edge cells as well. If the edge width was *n*, the wild area was expanded to include all the cells connected to any cells that had been considered as edge cells when width was *n* – 1. Throughout, any cells that did not count as an edge cell was considered as a cultivated field cell.

For all simulations, a viable nesting site was selected by randomly selecting a field edge cell within the middle 98×98 cells of the arena (this area was confined to accommodate the maximum radius of foraging considered). The numbers of edge (*e_vis_*, termed **‘visible wild’**) and cultivated (*c_vis_*) field cells whose centres lay within a pre-defined foraging radius of the nest cell's centre were counted, and the total number of cells visible was determined (as *n_vis_*  =  *e_vis_* + *c_vis_*). At the same time, the total number of edge (*e_tot_*) cells throughout the environment was calculated. The proportion of edge cells (**‘proportion wild’**) visible to a nesting forager was calculated as *e_vis_*/*n_vis_*. We also calculated a **‘coverage’** statistic that gave a description of how composition of the forager's available foraging environment compared to what was available throughout the entire 250,000 cells of the modelled environment: we did this by calculating the ratio of the proportion of locally visible cells that were edge cells to the overall environmental proportion was calculated as (*e_vis_*/*n_vis_*)/(*e_total_*/250,000).

For the simulations, 10,000 replicates were conducted for each systematically altered parameter, with other parameters being selected using a randomisation function. When randomised, foraging radius was a real number from the range (0, 200); field edge width was an integer value from the range (1, 15); and the number of initial field seeds was an integer value from the range (2, 200).

In addition to the separate exploration of the three individual model parameters, we also examined the interactions of these parameters by generating 200 fields (each with independently randomised initial seed coordinates) for each of the 512 possible combinations where foraging radius  =  (25, 50, …, 200), edge width  =  (1, 3, …, 15) and field seeds  =  (25, 50, …, 200). The changes in the calculated values of ‘visible wild’, ‘proportion wild’ and ‘coverage’ were modelled using an analysis of variance which incorporated the three parameters, the three possible two-way interactions and the single three-way interaction between them.

### Models using British landscape data

Areas of UK landscape were selected from land described within UK Ordnance Survey squares NN, NY, SE, SJ, SK, SO, SP, ST, SU and TL (these particular squares were chosen because they contained little or no sea), using the most current data available at a 1∶10,000 scale on the 19^th^ July 2010. To be acceptable as a representative of the British non-urban ‘landscape’, each valid square used could contain housing and associated small gardens on no more than 25% of the area. Similarly, squares were not deemed acceptable if they contained large bodies of water (lochs, lakes, or the sea). One hundred 2×2 km^2^ squares that fit these criteria were randomly selected for processing.

A traced outline of the field edges was made for each of the 100 samples, where streams, rivers, roads, walls, paths, and marked edges of wooded areas were considered to be field edges. This simplification is justified here as the areas selected for sampling were predominantly non-urban, and were intended to give an approximation of the form of the UK countryside rather than being an exact representation (the maps used could not give exact geographical information of the existing widths of many of the linear structures within the environment due to the scale of mapping available). The traced images were scanned, and converted to 813 × 813 best-quality JPG images using *Preview* 5.0.2 (Apple Inc., Cupertino, California, USA). These files were then converted to binary images using *ImageJ* 1.43*u* [57], and saved as text images.

As a result of this process, the unit squares in the 813×813 landscapes created in this manner were either ‘edge’ or ‘field’ cells. Therefore, the unit edges of each square considered corresponded to a geographical distance of 2.46 m (a constraint imposed by the pixel resolution of the scanned images – therefore, any geographical ‘edge’ feature as defined above was assumed to possess unit cell width). For each of these landscapes, the mean proportion of edge cells (relative to field cells) available to a forager nesting within the field margin that had a foraging radius of *r* units was assessed. To do this, all possible nesting sites within the landscape that were able to support this foraging distance were identified, and the numbers of edge and field cells were calculated for each of these. For example, the maximum radius of *r* = 406 could only be assessed if the central cell in the arena was an edge cell. A forager with a foraging radius of *r* = 405 could potentially nest in any of cells within the central 3×3 region of the arena, and if all of these were edge cells, the calculated mean proportion for *r* = 405 would be the mean for the nine foraging areas centred on these nine cells. For a forager with *r* = 1, the nest could potentially be located in any of the central 811 × 811 cells. A single mean value for all values of *r* between 1 and 406 was calculated (if possible) for each of the 100 landscapes assessed.

To assess the effects of field margin manipulations within these natural landscapes, the field edge cells as extracted above were manipulated by adding one, two or three extra layers onto them, using identical techniques to those described for the Voronoi-like edge width manipulations. The mean wild space available to foragers travelling *r* = 1 – 406 units were then assessed for these manipulated landscapes as described above.

## Results

### Voronoi-like randomised landscape

**Figure 2:**
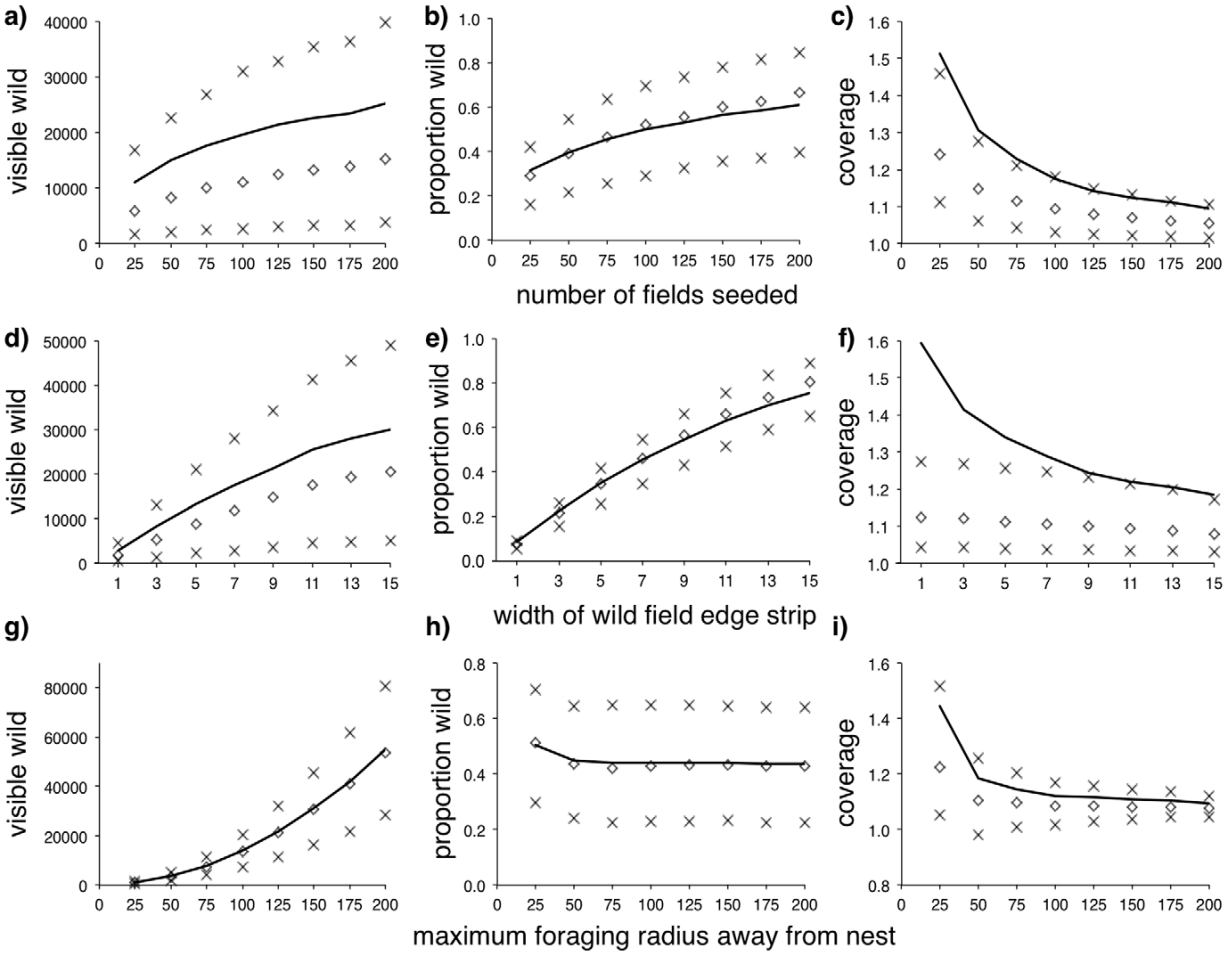
Wild forage available within Voronoi-like randomised fields. Solid black line gives mean values and symbols give the 25%, median and 75% interquartile values for the mean number of visible wild field edge cells (‘visible wild’, panels *a*, *d* and *g*) and the proportion of wild field-edge cells to cultivated field cells within the foraging radius (‘proportion wild’, panels *b*, *e* and *h*), and the ratio of the proportion of field edge cells visible within the foraging distance of the nest compared with the overall proportion of edge to field cells within the simulated arena (‘coverage’, panels *c*, *f* and *i*), where the number of fields seeded (panels *a*, *b*, and *c*), the width of the wild field edge strip (panels *d*, *e*, and *f*), or the radius of the foraging distance around the nest (panels *g*, *h*, and *i*) are systematically altered.

Increasing the number of field seeds used to generate the Voronoi landscapes would have increased the density of fields within the simulated environments, and we would therefore expect the amount of hedges available to increase, which we see in the increase in both wild space visible to the forager (Fig. 2*a*) and the proportion of the visible environment that was wild (Fig. 2*b*). If fewer field edges are available, the ratio of wild habitat that a forager experiences will be much greater than the overall ratio seen in the environment (Fig. 2*c*), because nesting within a field margin will mean that the forager has a disproportional amount of adjacent wild space available for it forage in. As the amount of edges increases, this relationship becomes much more representative of what we see on average within the environment.

Increasing the width of the wild field strip should have a similar effect, as more foraging area is made available to a forager who is already nesting within the wild area. This is reflected in the increase in visible wild habitat (Fig. 2*d*) and the proportion available (Fig. 2*e*), and the corresponding decrease in coverage as wild margin habitat becomes more available within the environment (Fig. 2*f*).

The radius over which a forager will forage away from its nest has obvious effects upon the amount of wild habitat available: increasing foraging distance increases the amount of wild space encountered (Fig. 2*g*). The proportion of the foraging area that is wild shows a different relationship however. With the exception of foragers who only travel a very small distance (who experience slightly more wild habitat because the immediate environment around the nest is very likely to contain field edge), there is negligible change in the proportion of wild habitat that foragers experience relative to the distance that they travel (Fig. 2*h*), suggesting that manipulations of margin availability should have similar effects upon foragers regardless of their commuting radius. The proportion of wild encountered relative to the amount actually seen within the environment only differs strongly for short-distance foragers (Fig. 2*i*), again suggesting that manipulations should have a scale-free effect with regard to foraging distance.

All the described relationships held within the additional datasets that were generated to explore the interactions between all three parameters. The relationships for each of the parameters considered separately were significant (Table 1). All the interactions between the terms were also significant (Table 1), and the trends revealed by these interactions closely followed the trends for individual parameters (Figure S1), with no differences in the qualitative patterns that emerged.

**Table 1:** Interactions between the three parameters considered in the Voronoi-like field generation model.

	degrees of freedom	visible wild	proportion wild	coverage
number of fields seeded (n)	7,101888	171934.85	56482.05	4045.19
width of edge strip (w)	7,101888	809334.96	307115.60	138.79
foraging radius (r)	7,101888	2251036.27	1632.33	2729.75
n × w	49,101888	4269.28	1375.74	2.00
n × r	49,101888	17354.86	179.16	558.71
w × r	49,101888	78643.72	37.09	15.14
n × w × r	343,101888	436.27	6.36	2.66

The table presents the *F* values, all of which are significant (*p*<0.001).

### British landscape

**Figure 3:**
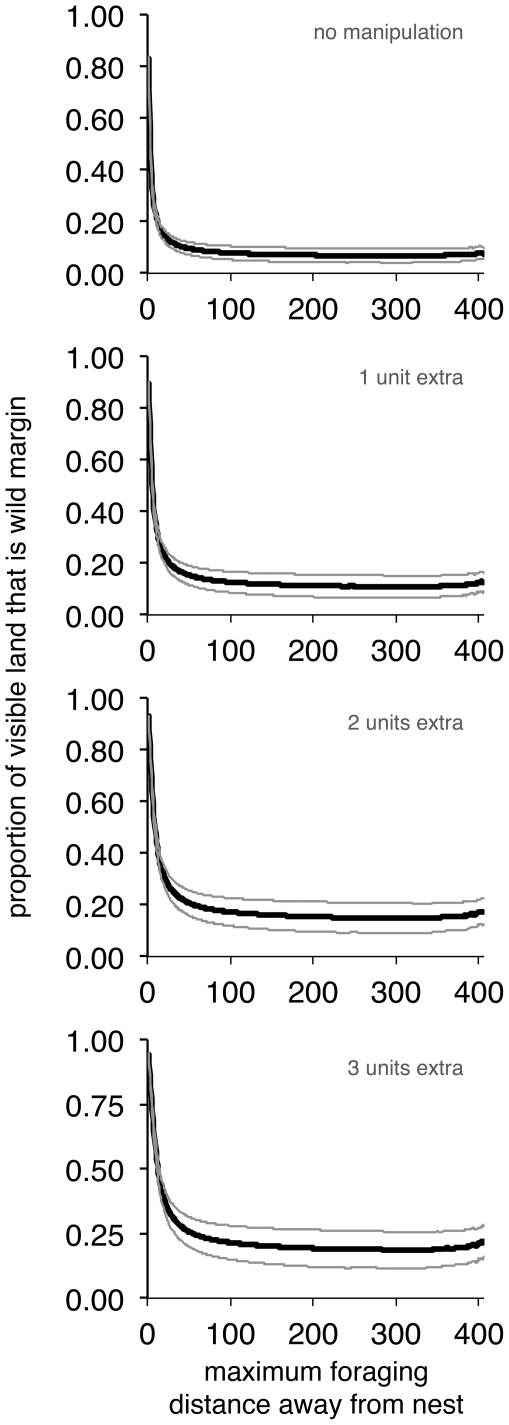
Results from the British landscape datasets. These panels show mean proportion (± s.d.) of wild field edges visible according to the foraging radius away from a nest, for (top to bottom) unmanipulated fields, and fields with an extension of 1, 2 and 3 units in their edge margin.

For both the natural environment and those where the field margins were expanded by one, two or three units (Figs. 3*a*-*d*), there is a similar relationship shown to the Voronoi-like results for foraging distance manipulation (Fig. 2*h*): only foragers travelling very small distances away from the nest are likely to see a large amount of wild habitat in which to forage. Once foragers are able to travel more than *c.* 50 units from the nest (corresponding to a real-world distance of about 125 m), the proportion of wild habitat available to them essentially becomes scale-free. The panels in Figure 3 show a slight increase for very large foraging distances, but this is more likely to be an artefact of the small number of samples that were possible for assessing these larger distances.

**Figure 4:**
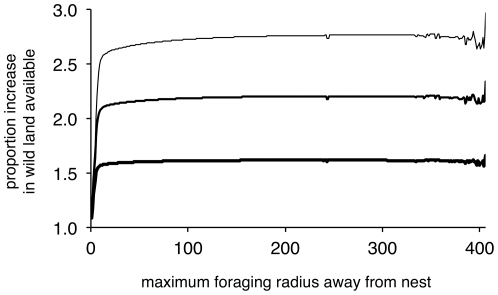
Increased availability of wild forage as a proportion of the ‘initial’ amount. Results for fields with edges manipulated by one (bottom), two (middle) or three (top) units, given as a proportion of the non-manipulated edge results.

Increasing edge width within the natural environments led to an increase in the proportion of wild habitat available to the forager, where field edges that were supplemented by three units showed the greatest proportion (Fig. 3*d*). This corresponds to the increase in the proportion of wild habitat available within Voronoi fields when the edge strip was widened (Fig. 2*e*). This is also visible when we directly compare the proportion of field edge available after manipulation with what is originally available (Fig. 4), where increasing width by a unit gives a corresponding increase in the extra proportion of wild space visible to a forager. Note also that this figure demonstrates the scale-free effect for foragers travelling more than about 50 units from the nest (and again, the noise for large foraging distances is likely to be a result of the small number of samples available).

## Discussion

Our models demonstrate that enhancing field margins should lead to a corresponding increase in the availability of forage to bees and other beneficial invertebrates that nest within the landscape. This was true for both the randomly-generated Voronoi-like fields, and for the landscapes extracted from the British data. With the exception of species that only forage within a very short range of their nest (less than 125 m), the effects of a given amount of field margin manipulation should enhance the proportion of land available to a pollinator for foraging regardless of the distance over which it normally travels to find food. Therefore, field edge manipulations should be beneficial for both longer-distance foragers such as honeybees, and short distance foragers such as solitary bees. Supplementing the resources available to the latter may well be essential for ensuring their survival within the agricultural environment, as it has been demonstrated that their ability to provision their brood declines as the distance they have to travel to find food increases [58]. Most species forage in an area greater than that bounded by the critical range estimate of 125m [47], but there may conceivably be a few short-ranging species that do not benefit from edge manipulations. For example, the threatened solitary mining bee *Andrena hattorfiana* has been in decline within the UK and Europe over recent decades [59], [60], and has a recorded maximum foraging distance of 130m from its nest [61]. Field edge manipulations would not be sufficient for this species if it did only forage within such a small area, and other forms of intervention would be required (in this particular case, perhaps targeting the environmental availability of this oligolectic species' principal foraging plant, field scabious *Knautia arvensis* [61]). This prediction of a critical distance is partly dependent upon the used British landscape data accurately representing what actually exists, and we would suggest that more detailed explorations are carried out with landscapes that accurately represent the foraging environment of species (such as *A. hattorfiana*), which would consider the availability of specific resources (such as field scabious) in the environment rather than just a general bank of ‘wild’ forage.

Here, we considered landscapes in two ways: using a randomly generated process, and extracted from existing landscape data. Although the two forms of landscape gave qualitatively similar predictions about changes in resources available to foraging pollinators, we need to be careful in considering how similar these two approaches are. Being able to randomly generate landscapes is a useful tool for considering the general effects of land use changes. Well-established techniques exist for modelling general landscape structure [62], [63], with some applied specifically to generating mosaic-like landscapes (*e.g.* [64]–[66]). Le Ber *et al*. [67] describe a platform that generates randomised field structures based on both rectangular and Voronoi-like tessellation processes, and demonstrated that neither technique perfectly simulates fields with a similar landscape structure to comparable French agricultural landscapes. How these generation processes match with other agricultural landscapes (such as the British case study used here) would need to be tested, but being able to easily generate landscapes to test different land use manipulations within is a useful tool for understanding landscape processes [67], and gives a simple tool for exploring how landscape structure may influence the behaviour of individuals [68], [69]. Although [67] suggests that the Voronoi-like fields are unlikely to match the exact landscape structure of any given environment, being able to generate any kind of field mosaic gives a means of exploring different environmental manipulations provided we acknowledge the limitations that may be caused by the lack of realism. We would suggest that the qualitative trends generated by this technique give us at the very least an indication of the direction of change of the processes we are interested in (as would the regular rectangular lattice arrangement we described in [49]).

This model, and especially the results using landscape structure derived from British maps, classified land as either wild or cultivated. Crops within cultivated agricultural fields may well provide valuable foraging resources to the forager as well, but are likely to only be available during a small timeframe within the year. Field margins should provide a diversity of resources that are available when crops are not. Of course, the model treats all wild land as being of equal worth, and maybe finer-scale differentiation for habitats such as stream edges and wooded habitat would give us insights about finer details of field edge manipulations, as habitats like these could offer different (and possibly richer) arthropod assemblages to hedgerows [70]. Furthermore, no differentiation was made for the presence of domestic gardens, which are increasingly seen as highly beneficial to foraging bees [71], and which were most probably present within the British landscapes used in this model. The presence of floral resource availability within the environment is absolutely critical to enhancing bee population size [72], and it could be argued that adding any form of wild land to the available foraging environment will therefore be seen as advantageous, although consideration may also need to be given to the general heterogeneity of the landscape [73].

Landscape structures may also have effects upon how pollinators forage through the environment, where hedges act as barriers for dispersal. Our model does not consider how field margins could act to impede forager dispersal here, but we do note that potential barriers such as thick forest may have little effect upon the ability of bees to move to foraging patches [39]. Nor do we consider how field edges may act as corridors [74], as we assume that bees will be scouting for food within the entire area around the nest, and constantly altering their foraging patterns to account for new sources [75].

Here, we specifically consider how changes in field edge structure could impact on the British landscape, which encompasses just a few of the many forms of agricultural environment found within Europe [76]–[79], many of which are becoming more fragmented with continued urbanisation [80]. Enhancing field edges is feasible within a heterogeneous agricultural landscape that consists of fields interspersed with hedges, but the applicability of these results to any agricultural landscape will depend upon the structure of the wild land within agricultural systems. Internationally, many landscapes do involve similar interconnected refuge areas [77]–[79], [81]–[85], and it may be fruitful to use the techniques described here to explore how their structure influences forage availability to native bees and other beneficial organisms that are constrained to return to a fixed point within the landscape. In the example we consider here, we took a heavily simplified approach to extract information about the availability of uncultivated areas within agricultural landscapes, and manipulated this to explore how resources could change with regard to simple changes in land use. We suggest that similar approaches could be used with more detailed landscape data (such as that extracted from land cover databases [76]). Making simple assumptions about changes in land use policy demonstrated that we could potentially consider the effects that regional policy could have upon pollinators with different foraging behaviours, and we would suggest that the techniques we develop here could be adapted to target both individual species (where the foraging biology is known) and individual locations. Furthermore, regardless of the amount of detail necessary to consider more specific cases, demonstrating that landscape manipulations are equally beneficial to nesting foragers that provide valuable ecosystem services regardless of their foraging geometry is an important consideration when justifying land being set aside from agricultural requirement.

## Supporting Information

Figure S1 **Two-way Interactions between parameters used in Voronoi-like field models**. Lines show the mean values of ‘*visible wild*’, ‘*proportion wild*’ and ‘*coverage*’, all as defined for Figure 2. ‘*n*’ represents the number of fields seeded, ‘*w*’ represents the width of the edge strip, and ‘*r*’ the foraging radius. The arrows give an indication of the direction of change for the parameter whose change is represented by the separate lines within each panel.
(PDF)
