# Supplementary figures and images for: Field Margins, Foraging Distances and Their Impacts on Nesting Pollinator Success
Source: PLoS One. 2011 Oct 3;6(10):e25971. doi: 10.1371/journal.pone.0025971 (PMC3185061; doi:10.1371/journal.pone.0025971)

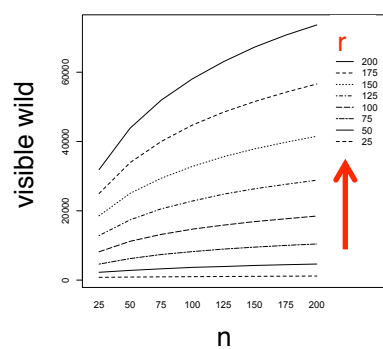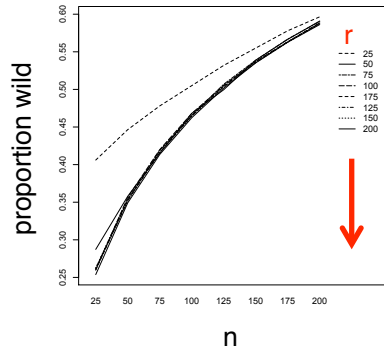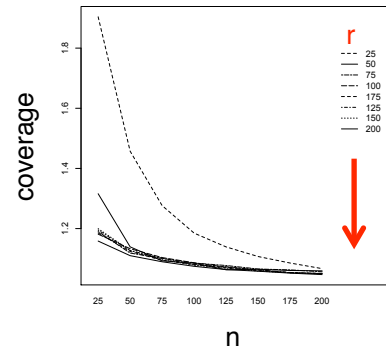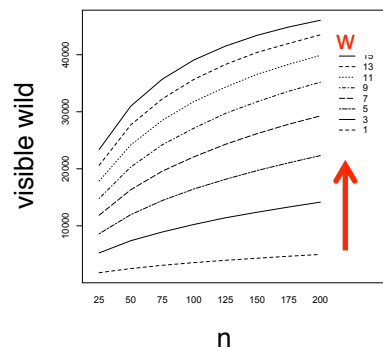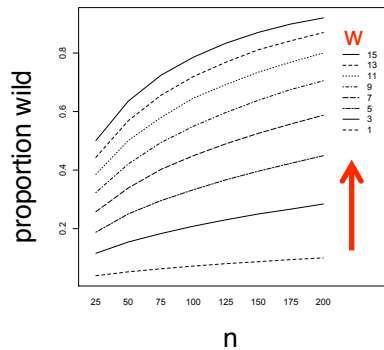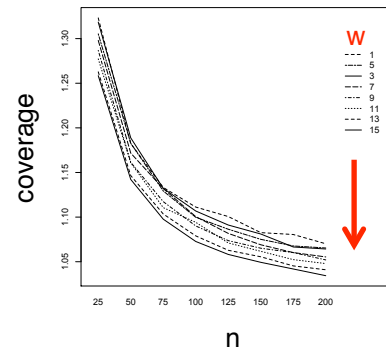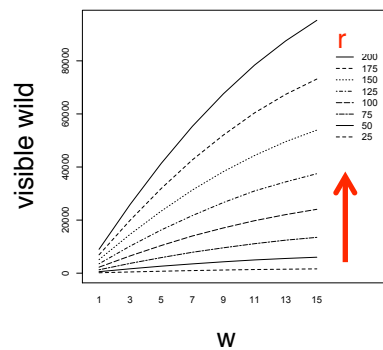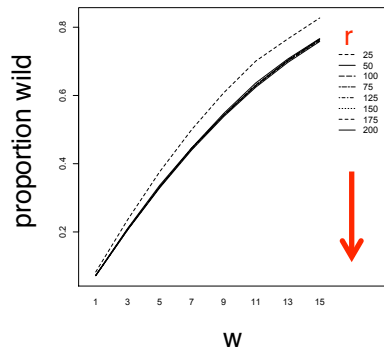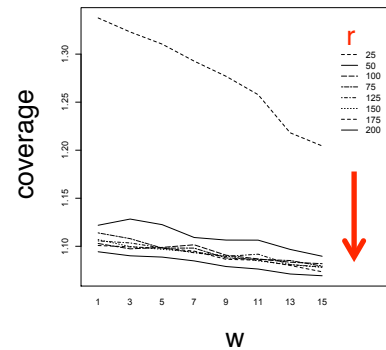

Supplement: Figure S1 — Two-way Interactions between parameters used in Voronoi-like field models. Lines show the mean values of ‘visible wild’, ‘proportion wild’ and ‘coverage’, all as defined for Figure 2. ‘n’ represents the number of fields seeded, ‘w’ represents the width of the edge strip, and ‘r’ the foraging radius. The arrows give an indication of the direction of change for the parameter whose change is represented by the separate lines within each panel. (PDF) [file pone.0025971.s001.pdf]
